# Personalized Medicine in Oral Oncology: Imaging Methods and Biological Markers to Support Diagnosis of Oral Squamous Cell Carcinoma (OSCC): A Narrative Literature Review

**DOI:** 10.3390/jpm13091397

**Published:** 2023-09-19

**Authors:** Dardo Menditti, Mario Santagata, Gianmaria Imola, Samuel Staglianò, Rita Vitagliano, Ciro Emiliano Boschetti, Angelo Michele Inchingolo

**Affiliations:** 1 Multidisciplinary Department of Medical-Surgical and Dental Specialties, University of Campania Luigi Vanvitelli, 80138 Naples, Italy; dardo.menditti@unicampania.it (D.M.); mario.santagata@unicampania.it (M.S.); gianmaria.imola@studenti.unicampania.it (G.I.); samuel.stagliano@studenti.unicampania.it (S.S.); ciroemiliano.boschetti@unicampania.it (C.E.B.); 2 Department of Interdisciplinary Medicine, University of Bari “Aldo Moro”, 70124 Bari, Italy; angeloinchingolo@gmail.com

**Keywords:** OSCC, oral carcinoma, imaging, diagnosis, prognosis, confocal microscopy, optical coherence tomography, ultrasounds, biomarkers, histology, metabolomic, microbiome

## Abstract

For decades, oral squamous cell carcinoma (OSCC) has been one of the most prevalent and mortal cancers worldwide. The gold standard for OSCC diagnosis is still histopathology but this narrative multidisciplinary review has the aim to explore the literature about conventional OSCC prognostic indicators related to the pTNM stage at the diagnosis such as the depth of invasion and the lymphovascular invasion associated with distant metastasis as indicators of poor life expectancy. Despite its multifactorial nature and recognizable precursors, its diagnosis at the early stages is still challenging. We wanted to highlight the importance of the screening as a primary weapon that a stomatologist should consider, intercepting all at-risk conditions and lesions associated with OSCC and its early stages. This narrative review also overviews the most promising imaging techniques, such as CT, MRI, and US-echography, and their application related to clinical and surgical practice, but also the most-investigated prognostic and diagnostic tissue and salivary biomarkers helpful in OSCC diagnosis and prognostic assessment. Our work highlighted remarkable potential biomarkers that could have a leading role in the future. However, we are still far from defining an appropriate and concrete protocol to apply in clinical practice. The hope is that the present and future research will overcome these limitations to benefit patients, clinicians, and welfare.

## 1. Introduction

The oral cavity may be affected by various malignant neoplasias, mainly of epithelial origin, such as oral squamous cell carcinoma (OSCC) and lymphoepithelial carcinoma [1], but also those arising from the derailment of oral melanocytes (oral melanomas) [2] and other soft tissues, such as sarcomas and angiosarcomas, adenocarcinoma, and adenocarcinoma not otherwise specified (NOS), as well as other rare tumors [3,4,5,6,7,8,9,10], and, on clinical bases, it is often difficult to distinguish them from benignant neoplasias and conditions.

To date, for OSCC, 377,713 new cases and 177,757 deaths have been estimated worldwide [11]. It can be anticipated by potentially malignant oral diseases (OPMDs) [12,13,14] and affects both sexes with a female/male ratio equal to 1:2.3 and mortality, registered in 2020, of 53,000 women and 125,000 men [15]. The most attributable risk factors are alcohol and tobacco consumption [16,17,18]. However, all those conditions responsible for hormonal changes, chronic injuries, deficiencies, and infections may also contribute to the tumor onset [19,20,21,22,23]. Lastly, UV radiation is the leading risk factor of lip cancer [24,25].

OSCC can arise from previously clinically healthy oral mucosa or on precursor lesions and conditions, previously classified as potentially malignant lesions and conditions, respectively, and these were recently reclassified as oral potentially malignant mucosal disorders or OPMDs. By definition, the precancerous lesion is a circumscribed oral mucosal lesion with a higher probability of malignant transformation than the mucosa surrounding the lesion; the precancerous condition is a generalized condition in which all the oral mucosa has more chance of malignant transformation than a subject without that condition. Last, OPMDs include conditions that affect the oral mucosa with an increased risk of malignancy, thus assuming, de facto, both precancerous lesions and conditions under the same group. Leukoplakia, erythroplakia, lichen planus, and submucous fibrosis are the most common OPMDs [12,13,14].

Some authors consider that various viruses have been associated with oral cancerogenesis [26,27,28,29,30,31]. Among them, HPV is historically and universally responsible for head and neck cancers of the oropharynx (OPC) and, as a matter of fact, from a clinical standpoint the evaluation of HPV-related markers is a valid parameter in the potential adjuvant or neoadjuvant treatment of oropharyngeal cancer regarding radiotherapy. In detail, HPV-16 is responsible for almost 90% of HPV-positive oropharyngeal cancers [32,33,34], mainly in men. Regarding the HPV-related cancers of the oral cavity proper, and thus excluding the OPCs, more than 97% of OSCCs are HPV-negative [27], and those that are positive could represent a subgroup of OSCCs of the base tongue [31]. Hence, HPV’s pathogenic role in OSCC is still debated. However, the literature reports a better prognosis in HPV-positive OSCC than HPV-unrelated OSCC, despite the finding that HPV in the oral mucosae is rarer than in the pharynx [26,33,35,36].

The HPV exerts its cancerogenic activity by integrating its genome with the host’s, thus allowing the expression of viral proteins, such as E6 and E7, which suppress keratinocytes’ expression of p53 and pRb, thus leading to immortalization. Furthermore, under predisposing conditions, cytokeratin 19, expressed by cancer cells, promotes, in a vicious circle, the production of HPV E7, thus allowing the progression of cancerization [33,34,35,37,38,39,40,41].

Furthermore, the role of bad oral hygiene and oral dysbiosis is still emerging [42,43]. In recent years, the knowledge of the oral microbiota associated with OSCC has been emerging regarding other pathogen microorganisms. Briefly, the microbiota, formerly known as “microbial flora”, is the community of microorganisms resident in specific body niches [44] whose dysregulation in composition and/or diversity, hence dysbiosis, may affect or be responsible for local and distant diseases [45,46,47,48], cancers included. The microbiota of the oral cavity changes by age, sex, and lifestyle [49,50,51] and varies according to local and systemic factors [52,53,54] and diseases [54,55,56,57]. Lastly, the role of chronic fungal superinfections is responsible for the detrimental conditions of oncologic patients, thus being a relevant risk factor for OSCC-related morbidity as well a risk factor for cancerogenesis itself [58,59,60,61,62,63,64,65,66].

The main issue related to OSCC is its late diagnosis, responsible for poor 5-year survival and substantial disabilities and bad health-related quality of life of OSCC survivors, who frequently may encounter recurrences or second primary tumors due to field cancerization [67,68,69,70,71,72]

To date, the conventional OSCC prognostic indicators are related to the pTNM stage at the diagnosis and mainly the depth of invasion and the lymphovascular invasion [1,73,74,75,76], both responsible for distant metastasis and poor life expectancy [77,78].

The recognition of OSCC at its early stage is possible through different measures related to the various degrees of prevention:

- Primary prevention (counselling at-risk subjects (stop smoking, stop drinking));
- Secondary prevention (screening and early diagnosis of OPMDs);
- Tertiary prevention (strict follow-up of OSCC survivors to intercept recurrences, metastasis, and/or second primary tumors).

In detail, screening is the primary weapon a stomatologist should consider when intercepting all at-risk conditions and lesions associated with OSCC and its early stages. In fact, due to its nonspecific clinical features at its earliest stages, OSCC is also called a “silent killer” and its diagnostic delay is a matter of life or death [79,80].

To potentiate the diagnostic procedures, various approaches can be considered.

To date, biopsy and histopathology are the gold standard for OSCC diagnosis. However, these procedures are invasive and need to be accurately planned, and the lesion to biopsy needs to be mapped through conventional imaging techniques, such as MRI and CT scanning, which are time-consuming and money-consuming procedures. Furthermore, these imaging systems may fail for early cancers and small lesions, and alternative approaches are necessary in these cases.

An adjunctive clinical approach consists of the use of noninvasive imaging systems that, bedside and in real time, can offer further details during the clinical examination of a suspicious lesion to shorten the time of diagnosis and orient toward appropriate management in less time, with more accuracy and less invasivity.

Hence, this narrative review aims to look at the main promising imaging techniques and overview the most-investigated tissue and circulating biomarkers—mainly focusing on those from saliva—indicative of cancerization or associated with the aggressiveness and severity of OSCC. Indeed, the markers’ predictive value to diagnose cancer and prognostic value will be reported.

## 2. Material and Methods

**Figure 1:**
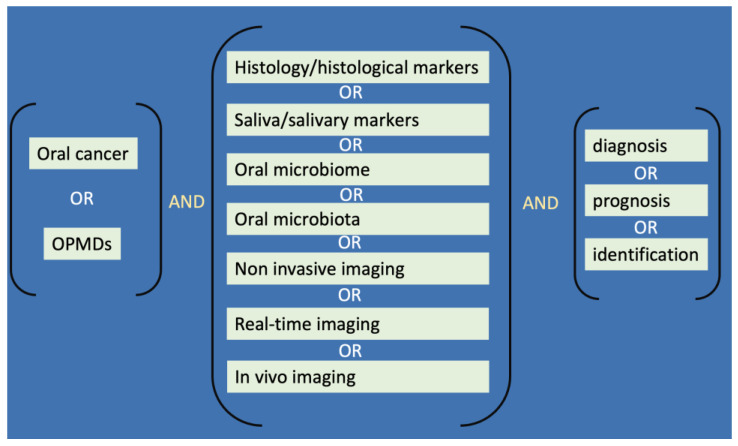
Sample scheme of the keyword search strategy.

The authors investigated a series of databases—Scopus, Web of Science, PubMed, and Google Scholar—for data extraction from scientific articles. The search ended on 10 February 2023. The selection included any kind of paper (original research, case reports, editorials, reviews) in the English language, dealing with any potential biological marker investigated in association with OSCC, OPMD, and controls, when present, without any limits in the range of years of publication. The keywords used for the search were as follows: oral cancer, OPMDs, markers, histology, saliva, oral microbiome, oral microbiota, imaging, noninvasive, real-time, diagnosis, prognosis, identification, and their synonyms, combined and matched by Boolean connectors (Figure 1).

The selected articles were grouped by topic and kind of marker studied. In this way, for convenience, the results have been presented in several sections, dealing, respectively, with imaging techniques—focusing on those innovative noninvasive and real-time ones—tissue markers, and salivary biomarkers—mainly adhesion molecules, enzymes, proteins, genes, various types of RNA, and metabolites involved in cancerogenesis or associated with cancer aggressiveness. Further research summarized the role of oral dysbiosis in the occurrence or maintenance of the OSCC microenvironment and reported the most-investigated microorganisms associated with OSCC onset and prognosis.

Mendeley Reference Manager software was used to export and manage the references.

## 3. Results

### 3.1. Imaging Techniques

Clinically similar conditions may underlie lesions utterly different from each other in nature and prognosis [81]. The possibility to benefit from imaging devices capable of noninvasively detecting signs of early cancer allows the strengthening of diagnosis parallel to less invasive treatments and better survival and quality of life. 

Among the most-investigated imaging tools in oral oncology, confocal microscopy (CM), optical coherence tomography (OCT), ultrasound echography (US-ECO), and tissue fluorescence (TF) [82] may be advantageously used to complement conventional RMN and CT scans [83] or to anticipate the diagnosis of small masses or early tumor changes unrecognized by these standard techniques. 

Briefly, confocal microscopy (CM) and optical coherence tomography (OCT) are based on an incident harmless laser source penetrating the epithelial layers. Then, an associated detector collects the backscattered co-planar or coherent light, respectively, thus allowing the digital rebuilding of virtual horizontal (in CM) or transversal (in OCT) sections of the tissue at a microscopic resolution [82,84,85,86].

Both CM and OCT applications in the imaging of the oral cavity are well documented in the literature [87,88,89,90,91].

Briefly, other than in ex vivo studies [92,93,94,95,96,97], CM has been variously applied in vivo, on human living oral sites, to virtually define histological signs of oral lichen planus and vesicular bullous oral diseases [98,99,100,101], to distinguish benignant lip pigmentations from melanomas [102,103,104], as well as to describe the dental enamel surface and its changes under peculiar conditions or treatments [105,106,107,108,109,110,111]. In oral oncology, CM demonstrated its capability to distinguish between suspicious oral lesions and OSCC with various histological degrees [112,113,114,115,116]. Furthermore, the literature reports similar experiences and success in OCT imaging on healthy oral mucosa [117], oral bullous diseases [118,119], and to discriminate OSCC cancers and their precursors [120].

Images similar to those gained by OCT imaging can be obtained by high-frequency US-echography, which is based on the transduction of sound waves backreflected from the tissue to a transducer capable of giving a visual image of the echoes, thus offering digital pictures of the body structures. In the oral field, this technology has been used to describe benign oral neoformations [121], as well as to characterize both healthy oral mucosa [121,122] and oral squamous cell carcinomas [123,124,125,126,127]. Furthermore, the high-frequency USs have been proven more accurate in estimating the thickness of thin carcinomas of the tongue when compared to MRI [128]. The potential application of high-frequency US-echography for the evaluation of the depth of invasion of OSCC is also an interesting point of reflection that is now emerging from a few studies. As a matter of fact, as reported in the eighth edition of the American Joint Committee on Cancer (AJCC) staging of oral cancer, the depth of invasion or DOI (≤5 mm, >5 mm but ≤10 mm, and >10 mm) in the new criteria has a fundamental role when categorizing a lesion from T1 to T4a and is strongly associated with neck node metastasis. US-echography, together with MRI and CT, could be very helpful in order to determine the preoperative DOI criteria and represent an important support for an adequate surgical preoperatory planning [129,130,131,132,133].

Other light-based imaging techniques useful in oral oncology are tissue autofluorescence [134,135,136,137], which can visualize the loss of emitted fluorescence typical of neoplastic lesions, and narrow-band imaging (NBI). NBI allows visualizing the architectural pattern of the intraepithelial papillary capillary loops of the vessels beneath the mucosa, whose shape and caliber blatantly differ among healthy mucosa, inflammatory conditions, and OSCC [138,139,140,141,142,143].

Each of the imaging techniques mentioned above has its advantages and limitations. Romano et al. [82] attempted to define the main functional characteristics of each one based on their capability to allow:

- Two-dimensional imaging (all techniques);
- Microscopic details of the tissues (RCM);
- Depth of invasion measurements (RCM, US, OCT);
- Tumor-associated neoangiogenesis and inflammation detection (NBI, RCM, US);
- Early signs of cancer detection (AF, RCM).
- Conversely, the main disadvantages and limitations can be ascribed to the following:
- Inapplicability to hyperkeratotic or melanocytic lesions (AF);
- Ergonomic limitation of the available devices (RCM);
- Costs and learning curve (RCM, US, OCT).

Table 1 summarizes the main findings about the noninvasive imaging tools mentioned above.

### 3.2. Tissue Markers

The most conventional approach to studying cancerogenesis considers genetic and epigenetic changes occurring in tumoral tissues. For this purpose, histological, immunohistochemical, and immunofluorescent studies focus on specific molecules, proteins, genes, or pathways differing from healthy normal cells. 

Being a stratified epithelium, the healthy oral mucosa histologically features the expression of cytokeratin types, peculiar to each layer and anatomical site, and each keratinocyte is well anchored and linked to the others by variously organized junctional proteins. 

Hence, a way through which an epithelial cancer cell may acquire motility and capability to metastasize is given when it loses the anchorage with other cells. Examples of altered expression of anchorage and cytoskeleton proteins involved in oral cancerogenesis are offered by alterations in cadherin family proteins and the interrelated proteins, such as integrins and vimentins [144,145,146,147]. E-cadherin alterations have been proven to provide cancer cells high propensity to invade and metastasize. This event is associated with its downregulation and delocalization from the membrane to the cytoplasm in cancer cells and is correlated to aggressive, poorly differentiated, high-grade OSCC and lower patient survival [148]. Furthermore, in a vicious circle, the delocalization of E-cadherin to cytoplasm seems due not only to the hypermethylation of its gene promoter, thus enabling E-cadherin synthesis, but also to the increase in epidermal growth factor receptor (EGFR) expression in OSCC that seems to favor E-cadherin internalization [148]. EGFR is, in fact, overexpressed in OSCC, which is associated with poor prognosis [149,150,151]. The evidence is such that several studies proposed anti-EGFR target therapies [152,153]. 

However, numerous other pathway dysregulations have been considered involved in OSCC, such as WNT/β-catenin pathway dysregulation. To better understand, it must be considered that, under physiological conditions, Wnt/β-catenin signaling regulates cell differentiation, proliferation, and apoptosis [154,155]. Hence, abnormal activation of this signaling promotes OSCC progression and metastasis [156]. The pivotal event leading to this dysregulation is caused by the aberrant expression of β-catenin which is not regulated by transmembrane WNT and can uncontrollably migrate to the nucleus, thus triggering a series of antiapoptotic WNT target genes, such as c-Myc, cyclin D1, and Bcl-2, thus upregulating proliferation and migration [157,158,159,160,161,162]. Furthermore, in these events, EGFR is also involved since it forms a complex with β-catenin and further promotes the cancer cell’s ability to invade and metastasize [163].

OSCC has also been proven to express higher than normal levels of cyclooxygenase type 2, conventionally associated with chronic inflammation [164,165,166]. 

Table 2 summarizes the evidence from the literature about the association between the most-investigated tissue markers and OSCC.

### 3.3. Circulating Markers

Since one of the goals of early diagnosis is the noninvasiveness of the procedure, another approach is offered by identifying biological markers from biological fluids, such as blood and saliva.

In detail, the use of saliva is convenient and noninvasive. Many studies reported hundreds of potential saliva biomarkers for OSCC and various targets identified by various methods. 

All the technologies used to identify and quantify these biomarkers are based on the so-called omics sciences: a term that has recently become widespread to comprehensively define each branch of molecular techniques focusing on specific targets [167]. By the definition given by Vailati-Riboni et al., the aim of omics sciences is “to identify, characterize, and quantify all biological molecules that are involved in the structure, function, and dynamics of a cell, tissue, or organism” [167]. 

In any case, a liquid biomarker can be investigated for diagnostic or prognostic purposes since some markers have been recognized as indicative of cancer (diagnostic value), while others are associated with the best or worst prognosis (prognostic values). In both cases, the presence/absence may be indicative, but its quantification (higher or lower expression) and the onset period (during the primary tumor stage, its recurrences or metastasis) should also be considered.

#### 3.3.1. Salivary Biomarkers

Concerning OSCC, its salivary biomarkers can be proteins, genes, various types of RNA, and metabolites. In each case, it must be considered that each molecule could be variously considered a diagnostic or prognostic marker. In the first case, the target-marker’s presence/absence or quantity indicates cancer; in the second case, its presence/absence or level of expression is associated with a better or worse life expectancy.

In 2022, Shaw et al. [168] meta-analyzed the scientific literature for the quantitative analysis of diagnostic accuracy and applicability of four classes of salivary biomarkers and the relative capability of polymerase chain reaction (PCR) and the enzyme-linked immunosorbent assay (ELISA) to detect them in the saliva. The authors reported results of detecting salivary biomarkers better by PCR than by ELISA; furthermore, the more sensitive and specific biomarkers associated with OSCC were mRNAs, miRNA, and IL-8 (sensitivity and specificity over 89%). Data were analyzed from over 1000 patients in thirteen eligible studies. 

In the field of omics sciences, the role of salivary proteins associated with OSCC has been widely investigated. Various systematic reviews and meta-analyses have recently summarized this research topic’s state of the art [168,169,170,171,172].

In detail, Arroyo et al. [172] analyzed the diagnostic capacity of salivary proteins as biomarkers for the differential diagnosis of OPMDs and OSCC compared to healthy controls. Their research was published in 2021 and considered, at the end of the selection, eight papers eligible for their purpose, thus reporting a series of potential markers, of which those significantly higher in OSCC/OPMDs than healthy controls, and therefore with diagnostic significance, were carcinoembryonic antigen (CEA) [173,174] and the soluble fragment of cytokeratin 19 (CYFRA21) [170,171]. In all these cases, *ELISA* was used as a helpful technique to discover and quantify proteins in a sample [175,176,177,178,179]. 

AlAli et al. [180] also found the prognostic significance of higher levels of IL-8 in cancer patients, whose overexpression, together with matrix metalloproteinase 9, MMP-9, was confirmed by other authors as associated with the worst prognosis. Later, Elmahgoub found the diagnostic significance of IL-1β, IL-6, and IL-8 for identifying early OSCC and OPMDs, as their levels were significantly higher than in healthy controls [181].

A similar approach to identifying the most significant diagnostic and prognostic salivary markers associated with OSCC and their different levels of expression varying during different stages of the disease was also reported by Riccardi et al. [171], who focused their systematic review and meta-analysis on a series of salivary proteins potentially helpful in screening oral neoplasia and predicting the prognosis of OSCCs. The authors grouped the main salivary OSCC biomarkers into five classes, such as cytokines (IL-1a, IL-1b, IL-6, IL-8, IL-10, TNF-α), other acute-phase response proteins associated with an inflammatory tumoral microenvironment (*AAT*a, HPX, C3, TTR, serotransferrin, and RETN), growth factors (vascular endothelial growth factor-a, VEGF-a), matrix metalloproteinases (MMP-1, MMP-2, MMP-3, MMP-9), and proline-rich proteins (PRPs) [171]. In detail, the prognostic role of cytokines like IL-1α, IL-6, IL-8, VEGF-a, and TNF-α was confirmed and remarkably accurate for tongue OSCC, with a poorer survival expectancy when overexpressed [182]. Furthermore, Riccardi et al. [171] summarized that early OSCC and OPMDs moving toward cancer exhibit overexpression of IL-6 and IL-1β. At the same time, TNF-α and IL-10 seemed to be more indicative of advanced OSCC, where they act by sustaining the inflammatory microenvironment where OSCC grows and acquires malignant and more aggressive behavior. 

Similarly, salivary levels of MMP-1 and MMP-3 have been proven to be up to 10-fold overexpressed in OSCC compared with healthy controls, and the increase seemed to be proportional to the OSCC stage, thus adding crucial prognostic information [183]. At the same time, MMP-2 and MMP-9 should be more indicative as screening biomarkers, peculiarly present in OSCC and those OPMDs moving toward malignant transformation [184,185]. Furthermore, among the proteins expressed in acute inflammation and cancer, there is broad evidence on how the inflammatory microenvironment is linked to cancer development and progression, mainly through the pathway involving COX-2 [164,165,186]. 

Moreover, among other kinds of salivary proteins investigated, resistin (RETN), a derived peptide hormone, seemed highly correlated with advanced OSCC and metastases, particularly in smokers and betel-chewers [187] and, similarly to other well-known hormones, such as the sexual ones, responsible for a gender-related difference in the onset and prognosis of specific subsets of OSCC [15,19,20]. 

The discovery of salivary biomarkers is accelerating from the advent and diffusion of other automated high-throughput techniques capable of massively sequencing genomes from tissues or fluids, known as next-generation sequencing (NGS). Used for sequencing both entire genomes and specific genetic sequences, NGS allows the identification of targeted sequences of cancer-related genes and RNAs. In the case of OSCC salivary biomarkers identified by NGS, the most-considered ones are genomic markers (genomic sequences indicative of genes involved in cancerogenesis) and proteomic markers, as described previously [188].

In the case of gene analysis, NGS aims to discover mutations at the gene level or methylation regarding promoter for oncosuppressor genes, known to silence oncosuppressor transcription, thus reducing their protein expression [148]. When used to find protein markers, NGS analyzes the transcriptomes (mRNA and small miRNA) to establish any relevant changes occurring in cancer. 

#### 3.3.2. Blood Markers and Circulating Cancer Cells

The various groups of “circulating markers” comprise a series of molecules—mainly tumoral cells, proteins, and nucleic acid compounds—whose presence in the blood has been proven indicative of OSCC at various stages. 

Recently, Zhu et al. [189] monitored, over a 5-year follow-up, the blood fibrinogen and albumin in OSCC patients to determine their prognostic value. In a cohort of over 150 patients suffering from OSCC, the increase in the fibrinogen-to-albumin ratio was reported to be an independent prognostic factor, negatively associated with the cancer-specific survival of the patients considered. These results are similar, and the evidence is reinforced, by the work published, in the same year, on bladder cancer by Barone et al. [190].

Other than inflammatory and nutritional circulating markers, other authors investigated the number and content of circulating extracellular vesicles carrying nucleic acids and proteins associated with OSCC. As reported by Brocco et al., extracellular vesicles (EVs) are “particles naturally delivered into the extracellular microenvironment, containing a rich cargo of DNA, RNA, miRNAs, proteins, lipids, and metabolites,” and they allow the transfer of their molecular cargoes, mediating short- and long-distance signaling, during physiological or pathological processes [191]. EVs are grouped by size and origin into exosomes, originating from the endosomal system and with a diameter of 30–150 nm, microvesicles, 100–1000 nm in diameter, released by plasma membrane budding, and apoptotic bodies, produced by apoptotic cells, with a variable diameter of 200–5000 nm [191]. Tumor cells have been proven to produce and release more EVs than healthy cells, and in cancer, they participate in various biological processes involved in tumoral neoangiogenesis, progression, and metastases [191]. Brocco et al. identified the EV concentrations and specific content from the peripheral blood samples of 106 oncologic patients and 25 healthy controls, thus proving the utility of EVs as predictors and diagnostic markers [191]. 

Furthermore, while the work by Brocco et al. focused on metastatic and locally advanced nonhematological cancer, Sun et al. [192] studied the EVs’ types by a proteomic approach, in salivary samples from 30 OSCC patients and 30 matched healthy controls, thus reporting the occurrence of 315 upregulated proteins and 132 upregulated phosphoproteins in OSCC. This work has been very recently published, and further developments are expected. 

Other than circulating proteins, both free or conveyed by vesicles, some researchers focused on cancer cells and circulating tumor nucleic acids, through a methodology known as “liquid biopsy”. This method focuses on the finding in body fluids, mainly plasma but also saliva, of potential biological markers associated with OSCC, like oncogenic microRNAs (miRNAs), small single-chain noncoding RNA molecules involved in mRNA inhibition for oncosuppressor proteins [193,194,195], circulating cell-free DNA (cfDNA), and mitochondrial DNA (mtDNA), both proportionally higher with OSCC aggressiveness and metastasis [196], or proper tumor cells. 

In this last case, the literature agrees with the finding that plasma-circulating cancer cells and cancer stem cells can be considered a valuable diagnostic and prognostic OSCC marker since their identification and amount are strictly and significatively associated with the TNM staging [197], with sensitivity, specificity, and accuracy globally over 95% [198].

### 3.4. Oral Microbiota Changes

A recent branch of oral oncology studies the oral microbiome and microbiota associated with OSCC. By definition, the microbiota is the microbic population specifically living in specific anatomical environments, such as the mouth and its niches, while the term “microbiome” refers to all genomic material distinct from the human genome [44,199]. 

Arising evidence has proven that specific changes in oral microbiota composition or oral microbiome expression occur associated with OSCC, as well proven for other extra-oral diseases and cancer [55,57,200,201,202].

Moreover, chronic oral infections, such as oral candidiasis and those sustaining periodontitis, also have been recognized as high-risk factors associated with the development of OSCC [15,60,64,203,204,205,206].

The most-investigated oral dysbiosis occurring in OSCC is associated with the overexpression of periodontopathogen bacteria such as *Fusobacterium nucleatum* and *Porphyromonas gingivalis*, responsible for chronic inflammation, bone resorption, hyperglycemia, and local and systemic toxicity, and an increase in oxidative stress resulting in overproduction of free radicals with oncogenic derailment [20,45,55,207,208]. These events can be directly and indirectly linked to bacterial proliferation and bacterial toxins, which may damage keratinocyte DNA beyond their repair capacities, thus allowing the accumulation of irreversible damage which leads to cancerogenesis. 

In general, oral dysbiosis is responsible for a microecological imbalance in the oral cavity and chronic inflammation and immune stimulations, which may trigger a stream of events that promote cancerogenesis [209].

In detail, *F. nuclatum* and *P. gingivalis* have been proven to enhance local inflammation at periodontal sites, thus exacerbating the production of those inflammatory cytokines previously described as associated with OSCC developments, mainly IL-6, *TNF-*α, and MMP-9, responsible for tissue dissolution and cancer progression, and proven to be overexpressed in OSCC [171,184,210].

Although the literature has reported the co-occurrence of oral dysbiosis in subjects suffering from OSCC, it is still unknown whether the bacteria anticipate and are directly responsible for OSCC or if peculiar dysbiosis occurs later, thus favored by the tumoral microenvironment. 

Some recent research has tried to respond to this question. 

Regarding the dysbiosis mechanisms involved in oral tumorigenesis, Pignatelli et al. [211] hypothesized three different mechanisms through which oral dysbiosis empowers oral tumorigenesis:

- the imbalance of keratinocyte proliferation and death;
- immune dysregulations;
- alterations of metabolisms of food compounds, drugs, and host metabolite.

Substantial efforts have been made to identify specific microbiota associated with OSCC.

For this purpose, as an example, Nie et al. [212] characterized the microbiota composition in different oral niches of OSCC in a sample of 65 OSCC patients. In their work, the microbiota identification of each niche in each subject was performed by 16S rDNA amplification and sequencing by PCR. Their results showed a noticeable difference in the microbiota composition of the tumoral niche compared with the neighboring and contralateral healthy areas. In detail, tumor tissue was peculiarly overcolonized by a greater abundance of *Fusobacteria*, Prevotella spp., Porphyromonas, Campylobacteria, Aggregatibacteria, Treponema, and Peptostreptococcus. Conversely, *Neisseria* and *Veillonella* were predominantly present in healthy tissues [212]. Interestingly, the species overexpressed at the tumoral sites were all anaerobic/facultative anaerobes. Furthermore, the authors associated the microbiota with clinical parameters, such as tumor size, histological tumor grading, TNM staging, and presence/absence of metastases, thus concluding on significant species predictive for metastases (but not for all the other indicators) by the significantly different identification and overexpression of *Parvimonas micra*, Prevotella pallens, Luteimonas marina, Peptostreptococcus stomatis, and Pyramidobacter piscolens [212].

These findings were shared by Pignatelli et al., who reported significantly higher levels of periodontitis-related taxa in OSCC subjects from the literature. Among the species most represented, the authors considered *P. gingivalis*, F. nucleatum, C. rectus, and P. stomatis, parallel to a significant decrease in Veillonellae [211].

Furthermore, most species were proven capable of metabolizing or fermenting host proteins into sulfides and nitrosamines, known as potent cell mutagens [213]. Among the most-reported species involved in this event, F. nucleatum, P. gingivalis, A actinomycetemconcomitans, and Prevotella intermedia produce the genotoxic and mutagenic agent hydrogen sulfide, responsible for chronic inflammation and cell migration and invasion, as well as cell proliferation and tumor angiogenesis [211]. 

Pignatelli et al. gave a further in-depth explanation of periodontopathogen bacteria with cancerogenic properties as due to the capability to produce volatile sulfur products responsible for the increase in ROS production and promoting collagen degradation of the basement membrane, thus favoring not only the cancer initiation but also invasion [211]. 

Various species have been considered able to interact with each other to amplify the oncogenic risk. This is the case for *F. nucletum*, which is capable of co-aggregating and interacting with various other bacteria, or *P. gingivalis*, which upregulates a series of genes responsible for loss of bacterial adhesion, thus reducing biofilm formation, as well as an increase in cell motility in tumor sites [214], so its salivary overabundance was proven to be correlated with advanced OSCC stages [215]. 

Otherwise, the acid production by *Lactobacilli* spp. seems to favor an acidic environment favorable to tumor growth, and their levels increase parallel to advancing TNM cancer stages [216]. Similarly, some Streptococcus spp. are associated with an increased risk of metastasis since they produce hydrogen peroxide and nitrogen dioxide, which are responsible for lowering pH and the development of hypoxia in the tumoral environment [217].

Lastly, the interaction between bacteria and alcohol or tobacco consumption must also be considered an additional factor in oral cancerogenesis. For example, *Neisseria* spp. are high producers of acetaldehyde. Hence, in an alcohol drinker, the alcohol consumed can be efficiently metabolized by these bacteria into acetaldehyde, of proven cancerogenic properties [218].

## 4. Discussion

As in past decades, OSCC is still one of the most prevalent and mortal cancers worldwide [1]. Despite the well-known multifactorial origin and the presence of recognizable and indicative precursors, its diagnosis at the early stages is still a challenge due to the unclear features at the initial stages and confounding factors or co-morbidities that can be underestimated by the subjects suffering this neoplasia.

In order to better identify the early signs of OSCC and prognosticate its behavior once diagnosed, OSCC can be approached by different technologies and methods.

This work has overviewed the current knowledge in the primary fields related to this issue, thus reporting the experiences from the scientific literature regarding noninvasive imaging techniques and diagnostic or prognostic markers of different natures and origins.

**Figure 2:**
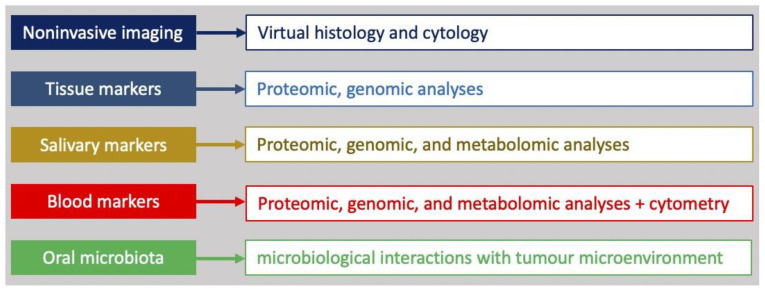
The potential analyses obtained by each methodology.

Table 3 and Figure 2 summarize the potential clinical support offered and the analyses obtained by each methodology, respectively.

Noninvasive imaging techniques showed the capability to support early and real-time appropriate diagnosis. Moreover, they could also guide toward the most appropriate therapeutical approach. Identifying a series of targeted molecules can be advantageous to indicate OSCC diagnosis and prognosis based on their presence or absence, increase or decrease, and structural or functional alterations associated with OSCC onset, recurrence, stages, or prognosis.

Among the imaging techniques, high-frequency ultrasound imaging, confocal microscopy, optical coherence tomography, and vascular imaging offered promising examples in the literature of advantageous helpfulness to defining, before surgery and before treatment, the nature of lesions suspected to be oral cancer.

Moreover, histological evaluation after surgery is still the gold standard for diagnosing OSCC, grading its histological disarray, staging its TNM features, and preliminarily hypothesizing a prognosis according to the classical parameters. In this context, scientists are constantly researching some tissue markers specific for OSCC, which could add further indications regarding the potential for malignant transformation in the case of OPMDs or that could be associated with aggressive forms of advanced OSCC.

However, the identification of tissue markers has a biopsy as a prerequisite and can be adopted only in the case of suspected lesions that require diagnostic insights through a conventional diagnostic pathway. Hence, the study of tissue markers is not applicable for screening programs or follow-up purposes.

In these latter cases, identifying other markers from fluids is more convenient; for this reason, scientists can draw on easily accessible bodily fluids such as blood and saliva. Leaving aside the blood, whose sampling is, in any case, invasive, the saliva remains, which is the ideal fluid, as it is easily accessible, collectible without invasive procedures, and in direct contact with any oral tumors or OPMDs. For these reasons, any changes in its composition can be easily detected, with simplicity and good patient compliance, giving reliable estimates of any metabolic or other changes in the course of oral carcinogenesis.

A salivary marker is each protein, RNA, or metabolite whose presence or absence or hypo- or hyperexpression is indicative of tumoral changes in the oral cavity. However, in any case, a universal marker may not be found due to heterogeneity across different ethnicities and populations [219].

For this purpose, a series of omics sciences have been developed and used to study the primary salivary markers with diagnostic or prognostic significance in oral oncology.

Among them, the study of the microbiome is the most recent omics science and studies the alteration in microbial, mainly bacterial, content, expression, and interaction, occurring during or before oral cancer development.

The investigations of all these kinds of molecules and bacteria shed light on the silent killer of OSCC and they could be considered, once further studies have concluded on the identification of more specific host molecules or bacteria, as indicative and noninvasive diagnostic and prognostic markers to accelerate diagnosis of early tumoral changes and predict the best therapeutical and follow-up approaches.

Furthermore, the improvement of the knowledge of the interaction between cancer and bacteria, as well as among chronic inflammation, cancer, and immune modulation, could lead in the future toward personalized approaches based on less invasive and more sensitive therapies, such as photodynamic therapy, gene therapy, probiotic therapy, engineered vaccines, or monoclonal antibodies [219,220,221,222,223,224,225,226,227,228,229,230,231,232,233,234,235].

Despite the arising knowledge, many mechanisms of action and interaction between bacteria and host and some crucial cancerogenic markers are yet to be identified.

Indeed, only a few studies have addressed the combinatorial value of these markers or performed statistical analysis to include interaction terms among oral dysbiosis, tissue changes, and salivary markers. These correlations may be considered through metabolomic studies, which were recently considered concerning OSCC. In this regard, the term “salivaomics” has been coined, and the interest in this branch has been exponentially increasing during the last few years [236,237].

Last, but not least, oral brush biopsy is another technique, less invasive than histology, to obtain crucial information through the cytology of the exfoliated cells from the oral mucosa. This modern and innovative technique is highly suitable for surgeons and well-tolerated by patients, as the collection of samples is usually painless and uneventful. The procedure includes the use of one or usually more brushes that are used by making a rotational movement for 10–15 s around the suspicious oral lesions or frank OSCC; it could be useful to also take a sample from healthy tissue or dysplastic tissue in order to make a comparison between normal and pathological tissue markers. Their collection from suspicious oral lesions or frank OSCC allows for analysis with various downstream methods depending on the marker to focus on and is widely versatile to analyze any cytological change in genetic content (DNA aneuploidies, altered mRNA expressions, methylation patterns, genes expression, and anomalies) as well in protein levels of expression or anomalies of location. For example, through a recent systematic review, Datta et al. investigated the effectiveness of DNA image cytometry (DNA-ICM) from brushings in differentiating OPMDs from benign/inflammatory lesions during screening and in predicting malignant transformation [238]. The authors concluded that there was still limited evidence in the literature about the success of DNA-ICM as an oral cancer screening tool and recommended further longitudinal and extensive community screening studies.

## 5. Conclusions

In conclusion, although each of the reported studies highlighted some potential prognostic and diagnostic biomarkers, we are still far from their advantageous use in clinical practice and, at present, none of the adjunctive tests reported and none of the biomarkers investigated can be recommended as a replacement for the current gold standard for diagnosis, which is still a surgical biopsy with the classical histological assessment.

We encourage further multicentric and comprehensive studies and hope that they will allow us to reach this critical goal by excluding a holistic approach and considering a multivariate analysis of the co-occurring biological, histological, metabolic, and microbiological changes occurring in cancerogenesis or proposing digital-supported analyses of big data or algorithms, through procedures or sample collections with low invasiveness. This is the case offered by the promising results from the study by The et al. [239], who recently assessed the quantitative Malignancy Index Diagnostic System (qMIDS) based on a multigene RT-qPCR method for the contemporary detection of a series of genes involved in cancerogenesis and immune dysregulations occurring during cancer, from tiny 1 mm^3^ minimally invasive biopsies.

## Figures and Tables

**Table 1:** The potential support in each step of OSCC management by each imaging tool.

Procedure	First Author (Year). Title. Country	Aims of the Study	Patients	Main Findings
Noninvasive imaging, reflectance confocal microscopy (RCM)	Contaldo et al. (2020). Intraoral Confocal Microscopy of Suspicious Oral Lesions: A Prospective Case Series. Italy [112]	To describe RCM cytoarchitectural findings in oral mucosae affected by OSCC and its precursors	Thirty oral sites in 21 patients with suspicious lesions; RCM vs. conventional histology	In vivo RCM was able to detect the main cytological and histological signs of oral malignancies Sensitivity, 1.000 Specificity, 0.933 PPV, 0.909 NPV, 1.000
Noninvasive imaging, reflectance confocal microscopy (RCM)	Peterson et al. (2019). Feasibility of a Video-Mosaicking Approach to Extend the Field-of-View For Reflectance Confocal Microscopy in the Oral Cavity In Vivo. USA [116]	To test a video-mosaicking approach to extend the field of view for intraoral RCM imaging	Oral sites from four healthy volunteers (normal oral mucosa), one patient (with an amalgam tattoo), and twenty OSCC patients were video-recorded by RCM to extend the field of view, compared with single-frame images	The video-mosaicking allowed appreciation of the whole lesions and their boundaries with a wider field of view than the classical single frames
Noninvasive imaging, fluorescent confocal endomicroscopy (FEM)	Abbaci et al. (2022). Diagnostic Accuracy of in Vivo Early Tumor Imaging from Probe-Based Confocal Laser Endomicroscopy versus Histologic Examination in Head and Neck Squamous Cell Carcinoma. France [114]	To assess the diagnostic performance of in vivo FEM in improving the management of early HNSCC	Forty-four patients with early head and neck lesions. FEM vs. conventional histology	Sensitivity, 73.2–75% Specificity, 30–57.4%
Noninvasive imaging, optical coherence tomography (OCT)	Yuan et al. (2022). Noninvasive Oral Cancer Screening Based on Local Residual Adaptation Network Using Optical Coherence Tomography. China [120]	To test a novel deep learning method for noninvasive oral cancer screening on OCT images	Twenty noncancerous OCT images and one hundred and forty-four cancerous images were considered from 25 patients	Accuracy, 91.62% Sensitivity, 91.66% Specificity, 92.58%
Noninvasive imaging, intraoral ultrasonography (ioUS)	Di Stasio et al. (2022). High-Definition Ultrasound Characterization of Squamous Carcinoma of the Tongue: A Descriptive Observational Study. Italy [123]	To describe the qualitative characteristics of tongue squamous cell carcinoma images obtained with high-definition intraoral ultrasound by comparing them with the corresponding histopathological sample	Twenty patients with tongue SCC were imaged by ioUS	Io-US was able to distinguish the tumor from the homogenous composition of the tongue tissues and the tumor margins
Noninvasive imaging, intraoral ultrasonography (ioUS)	De Koning et al. Application and Accuracy of Ultrasound-Guided Resections of Tongue Cancer (2022). The Netherlands. [125]	To test ultrasound (US)-guided surgical removal of squamous cell carcinoma of the tongue (SCCT)	Forty patients with SCCT underwent US-guided SCCT during surgery and the results were compared with ninety-six tongue cancer patients who had undergone conventional surgery	US-guided SCCT resections improve margin status and reduce the frequency of adjuvant radiotherapy. In the US cohort, the frequency of free margin status was significantly higher than in the conventional cohort, and the frequency of positive margin status was significantly lower
Noninvasive imaging, intraoral ultrasonography (ioUS)	Nilsson et al. Ultrasound Accurately Assesses Depth of Invasion in T1–T2 Oral Tongue Cancer (2022). Sweden [126]	To investigate the assessment of DOI using ultrasounds (US-DOI)	The DOI was assessed in 40 patients with T1–T3 SCCT by ultrasound, palpation, computed tomography, (CT) and magnetic resonance imaging (MRI). Histological DOI (pDOI) was gold standard	Ultrasound seems to be the most accurate method to assess DOI in T1–T2 SCCT. MRI overestimates DOI and cannot assess a substantial proportion of the tumors
Noninvasive imaging, intraoral ultrasonography (ioUS)	Caprioli et al. High-Frequency Intraoral Ultrasound for Preoperative Assessment of Depth of Invasion for Early Tongue Squamous Cell Carcinoma: Radiological–Pathological Correlations (2022). Italy [127]	To investigate the accuracy of ioUS in the assessment of the DOI in early OSCC (CIS, pT1, and pT2) compared with conventional histological DOI	Forty-one patients with tongue SCCs (CIS-T2) underwent a preoperative high-frequency US and the US-DOI was compared with the histological DOI (pDOI) and MRI-DOI	The ioUS was significantly accurate at determining the T stage ioUS sensitivity to predict a pDOI ≥ 4 mm, 92.31% ioUS specificity to predict a pDOI ≥ 4 mm, 82.14% ioUS specificity to predict an invasive cancer, 100% ioUS sensitivity to predict an invasive cancer, 94.7% ioUS NPV to predict an invasive cancer, 60% ioUS PPV to predict an invasive cancer, 100%
Noninvasive imaging, intraoral ultrasonography (ioUS)	Nogami et al. (2022). The Accuracy of Ultrasound and Magnetic Resonance Imaging for Estimating Thickness of Oral Tongue Squamous Cell Carcinoma and Influence of Biopsy on Those Findings. The Netherlands [128]	To compare the accuracy of MRI and ioUS to estimate DOI compared with their histological DOI (pDOI)	Eighty-three patients with tongue cancer underwent MRI and one hundred and seven ioUS. All MRI-DOIs and ioUS-DOIs were compared with their pDOI	ioUS-DOI is close to the pDOI in tongue cancers with DOI up to 10 mm. ioUS tends to underestimate DOI in tumors > 10 mm DOI. MRI tends to overestimate DOI in both thin and thick tumors
Noninvasive imaging, intraoral ultrasonography (ioUS)	Yesuratnam et al. Preoperative evaluation of oral tongue squamous cell carcinoma with intraoral ultrasound and magnetic resonance imaging-comparison with histopathological tumour thickness and accuracy in guiding patient management (2013). Australia [131]	To compare the accuracy of MRI and ioUS to estimate tumor thickness (TT) compared with their histological TT (pTT)	Eighty-eight patients with the presumptive diagnosis of invasive tongue SCC were analyzed. Seventy-nine patients had preoperative US and eighty-one had MRI	ioUS-TT demonstrated high correlation and MRI-TT moderate correlation with pTT
Noninvasive imaging, narrow-band imaging (NBI)	Contaldo et al. (2017). Evaluation of the Intraepithelial Papillary Capillary Loops in Benign & Malignant Oral Lesions by in Vivo Virtual Chromoendoscopic Magnification: A Preliminary Study. Italy [138]	To establish NBI’s feasibility to visualize and distinguish the intraepithelial papillary capillary loop (IPCL) patterns of benign oral pathologies from malignant ones	Benign lesions or OSCC from thirty-one patients were imaged by NBI before surgery and the IPCL classified according to their features into a four-class system	IPCL type IV was found only in malignancies Sensitivity, 100% Specificity, 93% PPV, 67% NPV, 100%
Noninvasive imaging, narrow-band imaging (NBI)	Nair et al. Narrow Band Imaging Observed Oral Mucosa Microvasculature as a Tool to Detect Early Oral Cancer: An Indian Experience. India [140]	To compare the capability of NBI and white light in defining the nature of clinically suspicious lesions	Fifty patients with suspicious malignant/premalignant lesions underwent white light imaging (WLI) and NBI to assess their IPCLs and were then compared with the standard histopathology	NBI sensitivity, 92.67% NBI specificity, 90.16% NBI PPV, 92.56% NBI NPV, 91.67% Accuracy, 92.16% The NBI group had a significantly better sensitivity and specificity with white light
Noninvasive imaging, narrow-band imaging (NBI)	Ota et al. Diagnostic Accuracy of High-Grade Intraepithelial Papillary Capillary Loops by Narrow Band Imaging for Early Detection of Oral Malignancy: A Cross-Sectional Clinicopathological Imaging Study. Japan [141]	To clarify the advantages and disadvantages of conventional visual inspection (CVI), endoscopic white light imaging (WLI), and narrow-band imaging (NBI) and to examine the diagnostic accuracy of intraepithelial papillary capillary loops (IPCLs) for the detection of OSCC	Sixty participants with oral mucosal diseases suspected of having oral potentially malignant disorders (OPMDs) or OSCC underwent CVI, WLI, NBI, and incisional biopsy. Images were evaluated to assess the lesion size, color, texture, and IPCLs	NBI accuracy, 100% NBI sensitivity, 80.9% NBI specificity, 59.1% NBI PPV, 100% NBI NPV, 85% NBI was negatively influenced by mucosal thickness
Noninvasive imaging, narrow-band imaging (NBI)	Sachdeva et al. (2022). A Prospective Study to Evaluate the Role of Narrow Band Imaging and Toludine Blue in the Screening of Premalignant and Malignant Lesions of the Oral Cavity in a Tertiary Referral Centre. India [142]	To compare NBI and toluidine blue (TB) for OSCC screening	Forty-four patients with suspicious oral cavity lesions (premalignant and malignant) underwent NBI and TB screening	TB sensitivity, 66.6% TB specificity, 87.8% NBI sensitivity, 66.6% NBI specificity, 95%. Malignant lesion sensitivity and specificity of toluidine blue were 94.3%, 100%, while the same for NBI were 100%, 88.8%, respectively. Both NBI and toluidine blue staining can be adopted for screening and the accurate detection of biopsy site and in the follow-up of premalignant lesions to look for malignant transformation
Noninvasive imaging, narrow-band imaging (NBI)	De Wit et al. (2022). Comparison of Narrow Band and Fluorescence Molecular Imaging to Improve Intraoperative Tumour Margin Assessment in Oral Cancer Surgery. the Netherlands [143]	To compare NBI with fluorescence molecular imaging (FMI), to study which intraoperative technique best assesses the mucosal tumor margins	Sixteen patients were halved into a group receiving NBI and another FMI. FMI was an ex vivo procedure, after patients intravenously received cetuximab, two days before surgery	Ex vivo FMI performed more accurately than in vivo NBI in mucosal margin assessment, mainly because NBI cannot detect submucosal extension. NBI adequately identified the mucosal margin especially in early-stage and not previously irradiated tumors

**Table 2:** The tissue markers mainly associated with OSCC.

Marker, Clinical Relevance	First Author (Year). Title. Country	Aims of the Study	Methods	Main Findings	Conclusions
E-cadherin, vimentin prognostic relevance	Puneeta et al. (2022). Evaluation of E-Cadherin and Vimentin Expression for Different Grades of Oral Epithelial Dysplasia and Oral Squamous Cell Carcinoma—An Immunohistochemical Study. India [144]	To evaluate the expression of vimentin and E-cadherin in different grades of oral epithelial dysplasias (OEDs) and oral squamous cell carcinoma (OSCC)	Immunoistochemical (IHC) analysis of E-cadherin and vimentin expression in H&E-stained specimens from 5 normal oral mucosa, 60 oral epithelial dysplasias (OEDs), and 60 different grades of OSCC	In OED, downregulation and altered localization of e-cadherin (81.6% of cases) In OED, increased expression of vimentin (52.3% of cases) In OSCC, reduction in expression (<50%) of E-cadherin (56.6% of cases) and altered localization (88.3% of cases) of E-cadherin associated with neoexpression of vimentin in the epithelial cells (68.3% of cases)	The E-cadherin downregulation and vimentin neoexpression in epithelial cells are indicators of progression from OED to OSCC and OSCC’s potential to metastatize
E-cadherin diagnostic relevance	Khan et al. (2022). E-Cadherin as a Prognostic Biomarker in Oral Squamous Cell Carcinoma: A Pilot Study at Tertiary Care Hospital. India [145]	To investigate the expression of E-cadherin in different grades and stages of OSCC and to elucidate its role as a reliable and potential marker	Immunoistochemical (IHC) analysis of E-cadherin and vimentin expression in H&E-stained specimens from 50 specimens of OSCC	Well- and moderately differentiated OSCC showed cytoplasmic and membranous E-cadherin Poorly differentiated OSCC, showed E-cadherin localized in cytoplasm The quantitative E-cadherin expression significantly decreased in poorly differentiated OSCC	OSCC shows E-cadherin delocalized in cytoplasm. E-cadherin expression depended on histological grading of OSCC. It was also found that as the tumor grade increased, there was decrease in membranous positivity. However, there was no correlation with the stage of disease
P-cadherin prognostic relevance	Lo Muzio et al. (2005). P-Cadherin Expression and Survival Rate in Oral Squamous Cell Carcinoma: An Immunohistochemical Study. Italy [146]	To assess the prevalence of P-cad expression in oral squamous cell carcinoma (OSCC) and to verify whether P-cad can be considered a marker of prognosis in patients with OSCC	Immunoistochemical (IHC) analysis of E-cadherin and vimentin expression in H&E-stained specimens from 67 specimens of OSCC	55.2% of OSCC showed membranous/cytoplasmic positivity for P-cad; 44.8% were negative Among P-cad +ve patients the best prognosis was for those OSCCs with membranous (*p* < 0.0001) compared to cytoplasmic P-cad expression	It is possible to suggest P-cad as an early marker of poor prognosis. The abnormal or lack of P-cad expression could constitute a hallmark of aggressive biological behavior in OSCC
E-cadherin prognostic relevance	Pannone et al. (2014). The Role of E-Cadherin down-Regulation in Oral Cancer: CDH1 Gene Expression and Epigenetic Blockage. Italy [148]	To investigate E-cadherin gene (CDH1) promoter methylation status in OSCC and its correlation with E-cadherin protein expression, clinicopathological characteristics, and patient outcome	Histologically proven OSCC and paired normal mucosa were analyzed for CDH1 promoter methylation status and E-cadherin protein expression by methylation-specific polymerase chain reaction and immunohistochemistry. Co-localization of E-cadherin with epidermal growth factor (EGF) receptor (EGFR) was evidenced by confocal microscopy and by immunoprecipitation analyses	E-cadherin downregulation and delocalization from the membrane to the cytoplasm in cancer cells is correlated to aggressive, poorly differentiated, high-grade OSCC and lower patient survival. Protein downregulation appeared to be due to E-cadherin mRNA downregulation and CDH1 promoter hypermethylation	Low E-cadherin expression is a negative prognostic factor of OSCC and is likely due to the hypermethylation of CDH1 promoter. The delocalization of E-cadherin from membrane to cytoplasm could be also due to the increased expression
Integrins and ECM components	Giannelli et al. (2001). Altered Expression of Integrins and Basement Membrane Proteins in Malignant and Pre-Malignant Lesions of Oral Mucosa. Italy [147]	To study the expression of integrins and ECM components (laminin-1, laminin-5, and collagen IV) of the BM in OSCC and OPMDs	IHC analysis on frozen specimens of OSCC, leucoplakia, and oral lichen planus	In OSCC, integrins’ polarity and distribution were altered. Ln-1, Ln-5, and Coll IV were discontinuous and interrupted in invasive SCC, whereas they were normal in the in situ carcinoma. In both premalignant lesions and lichen planus specimens, integrins were expressed in a polarized manner in the presence of a normal BM, whereas they were abnormally distributed in those tissues with altered staining patterns of the ECM components	Abnormal redistribution of integrins and expression of ECM components such as Ln-5 could play an important role in SCC invasion and metastasis
EGFR	Chung et al. (2006). Increased Epidermal Growth Factor Receptor Gene Copy Number Is Associated with Poor Prognosis in Head and Neck Squamous Cell Carcinomas. USA [149]	To establish if high epidermal growth factor receptor (EGFR) gene copy number is associated with poor prognosis in HNSCC	GFR status was analyzed in 86 tumor samples from 82 HNSCC patients by fluorescent in situ hybridization (FISH) to determine EGFR gene copy number, by polymerase chain reaction and direct sequencing for activating mutations, and by DNA microarray and immunohistochemistry for RNA and protein expression	58% of samples with FISH results demonstrated EGFR high polysomy and/or gene amplification The FISH-positive group was associated with worse progression-free and overall survival	High EGFR gene copy number by FISH is frequent in HNSCC and is a poor prognostic indicator. Additional investigation is indicated to determine the biologic significance and implications for EGFR inhibitor therapies in HNSCC
WNT pathway	Pannone et al. (2010). WNT Pathway in Oral Cancer: Epigenetic Inactivation of WNT-Inhibitors. Italy. [156]	To demonstrate the role of WNT pathway activation irrespective of beta-catenin mutation in oral cancerogenesis	Methylation-specific PCR (MSP) was used to study the methylation status of a complete panel of genes (SFRP-1, SFRP-2, SFRP-4, SFRP-5, WIF-1, DKK-3) involved in WNT pathway in thirty-seven cases of formalin-fixed, paraffin-embedded OSCC with relative controls of normal oral epithelium	SFRP-2, SFRP-4, SFRP-5, WIF-1, DKK-3 revealed methylation status of their promoter in OSCC, whereas SFRP-1 showed demethylation in cancer Statistically significant association between SFRP-2, -4, -5 gene methylation and OSCC	Epigenetic activation of some genes may activate WNT pathway in oral cancer
c-Met	Lo Muzio et al. (2004). Scatter Factor Receptor (c-Met) as Possible Prognostic Factor in Patients with Oral Squamous Cell Carcinoma. Italy [160]	To study the biological role of c-Met in oral tumorigenesis	IHC analysis on seventy-three cases of OSCC and ten of normal mucosa for c-Met expression	78% of OSCCs showed immunopositivity Among positive tumors, well-differentiated areas showed low or absent cytoplasmic positivity, while poorly differentiated areas showed both membranous and cytoplasmic positivity The frequency of lymph node metastases was higher in c-Met-positive tumors than in c-Met-negative ones Patients with negative/reduced c-Met expression had significantly better survival rates than patients with high expression	c-Met expression may be useful to identify cases of oral squamous cell carcinoma with a more aggressive and invasive phenotype
Bcl-2, c-Myc	Pallavi et al. (2018). Bcl-2 and c-Myc Expression in Oral Dysplasia and Oral Squamous Cell Carcinoma: An Immunohistochemical Study to Assess Tumor Progression. India [161]	To assess the expression of Bcl-2 and c-Myc in OED and OSCC	Thirty OEDs, thirty OSCCs, and ten normal gingiva were immunohistochemically assessed for Bcl-2 and c-Myc distribution, intensity, percentage of positive cells, localization, and immunoreactive scores	OED Bcl-2 positivity, 60% OSCC Bcl-2 positivity, 37% c-Myc showed 87% and 90% positivity within dysplasia and OSCC, respectively. In OSCC, c-Myc showed moderate intensity (*p* = 0.04). Average percentage of positive cells expressing c-Myc and Bcl-2 increased proportionally within grades of dysplasia (*p* = 0.000 and *p* = 0.008, respectively), whereas in OSCC, only c-Myc showed significant expression (*p* = 0.021). Localization of c-Myc was seen in the nucleus among OSCCs (*p* = 0.01). c-Myc and Bcl-2 showed moderate immunoreactivity in dysplasia (*p* = 0.005 and *p* = 0.013, respectively), whereas in OSCC, moderate immunoreactivity of c-Myc (*p* = 0.05) was observed	Variable expression of c-Myc and Bcl-2 reveals that these proteins act in synergism in early phases of carcinogenesis, whereas in later stages, due to the diminished activity of Bcl-2, c-Myc interacts in coordination with other oncogenes contributing to tumor progression
Bcl-2	Lo Muzio et al. (2003). Expression of Bcl-2 in Oral Squamous Cell Carcinoma: An Immunohistochemical Study of 90 Cases with Clinico-Pathological Correlations. Italy [162]	To explore Bcl-2 immunoreactivity in oral cancers and to assess its potential clinicopathological implications	Ninety OSCC and ten normal mucosal formalin-fixed, paraffin-embedded samples were analyzed for Bcl-2 expression by immunohistochemistry	Normal oral mucosa showed a cytoplasmic pattern of Bcl-2 immunoreactivity in the basal cell layers 83% of OSCCs showed no immunoreactivity 17% of OSCCs manifested consistent cytoplasmic positivity Patients with absent or low Bcl-2 in immunoreactive OSCC manifested poorer overall survival rates	Patients with absent or low (scores 0 and 1) Bcl-2 in immunoreactive tumors manifested poorer overall survival rates
Cyclooxygenase type 2 (COX-2)	Pannone et al. (2004). Cyclooxygenase-2 Expression in Oral Squamous Cell Carcinoma. Italy [164]	To analyze the expression of COX-2, at the protein level, in OSCC	IHC analysis on 45 OSCC specimens to define the COX-2 expression	77.8% of OSCCs showed moderate to high COX-2 expression **82%** of cases also showed intense COX-2 staining in endothelial cells of intra-tumor vessels and extra-tumor vessels adjacent to the tumor nests	**C**OX-2 is overexpressed in OSCC
Cyclooxygenase type 2 (COX-2)	Pannone et al. (2007). Cyclooxygenase Isozymes in Oral Squamous Cell Carcinoma: A Real-Time RT-PCR Study with Clinic Pathological Correlations. Italy [165]	To measure COX-1 and COX-2 mRNA expression in samples	COX-1 and COX-2 mRNA expression was measured by RT-PCR in 22 patients (OSCC and contralateral healthy mucosa)	A significant inverse relationship was found between COX-1 and COX-2 in each sample Higher levels of COX-2 expression were significantly associated with poor disease-free survival	COX-1 may play a role in oral carcinogenesis and could be regarded as a potential therapeutic target by chemopreventive drugs; moreover, COX-2 expression might be addressed as a new prognostic tool in the clinical management of OSCC

**Table 3:** The potential support in each step of OSCC management by each adjunct.

	Screening/Diagnosis	Prognosis	Follow Up
Noninvasive imaging	Real-time, surgery-free identification of histological and cellular abnormalities; tumoral neoangiogenesis evaluation; tumor thickness and behavior (infiltrating vs. growing)	Some parameters (cellular pleomorphisms, haploidies, depth of invasion, tumoral vascularization) identified through these methods can correlate with prognostic indicators of OSCC (metastases, survival)	Real-time, surgery-free, multiple control of healing or recurrences
Tissue markers	Invasive (requires biopsy) identification of molecules over- or hypoexpressed in OSCC. Not affordable for follow-up purposes (it requires further biopsies)		
Circulating markers (salivary and plasma) and liquid biopsy	Noninvasive (from saliva) or less invasive (from blood) identification and measurement of molecules and circulating tumor cells and tumor DNA predictive of OSCC	Noninvasive (from saliva) or less invasive (from blood) identification and measurements of molecules and circulating tumor cells and tumor DNA associated with prognostic indicators of OSCC (metastases, responsiveness to treatments, survival)	Noninvasive (from saliva) or less invasive (from blood) identification and measurements of molecules and circulating tumor cells and tumor DNA associated with OSCC recurrence or occult metastases
Oral microbiota	Identification, characterization, and quantification of microbiota composition peculiar to the tumor microenvironment before OSCC diagnosis, associated with prognostic indicators and for follow-up purposes		

## Data Availability

Data are available upon reasonable request from the corresponding author (R.V.).
